# Development of Y_2_O_3_ Dispersion-Strengthened Copper Alloy by Sol-Gel Method

**DOI:** 10.3390/ma15072416

**Published:** 2022-03-25

**Authors:** Jiangang Ke, Zhuoming Xie, Rui Liu, Ke Jing, Xiang Cheng, Hui Wang, Xianping Wang, Xuebang Wu, Qianfeng Fang, Changsong Liu

**Affiliations:** 1 Key Laboratory of Materials Physics, Institute of Solid State Physics, Hefei Institutes of Physical Science, Chinese Academy of Sciences, Hefei 230031, China; jiangang@mail.ustc.edu.cn (J.K.); zmxie@issp.ac.cn (Z.X.); jingke@mail.ustc.edu.cn (K.J.); cx2430@mail.ustc.edu.cn (X.C.); hwang18@mail.ustc.edu.cn (H.W.); xpwang@issp.ac.cn (X.W.); xbwu@issp.ac.cn (X.W.); csliu@issp.ac.cn (C.L.); 2 Scinece Island Branch, Graduate School, University of Science and Technology of China, Hefei 230026, China

**Keywords:** copper, sol-gel, mechanical properties, thermal stability, thermal conductivity

## Abstract

In this study, oxide dispersion-strengthened Cu alloy with a Y_2_O_3_ content of 1 wt.% was fabricated through citric acid sol-gel synthesis and spark plasma sintering (SPS). The citric acid sol-gel method provides molecular mixing for the preparation of precursor powders, which produces nanoscale and uniformly distributed Y_2_O_3_ particles in an ultrafine-grained Cu matrix. The effects of nanoscale Y_2_O_3_ particles on the microstructure, mechanical properties and thermal conductivity of the Cu-1wt.%Y_2_O_3_ alloy were investigated. The average grain size of the Cu-1wt.%Y_2_O_3_ alloy is 0.42 μm, while the average particle size of Y_2_O_3_ is 16.4 nm. The unique microstructure provides excellent mechanical properties with a tensile strength of 572 MPa and a total elongation of 6.4%. After annealing at 800 °C for 1 h, the strength of the alloy does not decrease obviously, showing excellent thermal stability. The thermal conductivity of Cu-1wt.%Y_2_O_3_ alloy is about 308 Wm^−1^K^−1^ at room temperature and it decreases with increasing temperature. The refined grain size, high strength and excellent thermal stability of Cu-1wt.%Y_2_O_3_ alloys can be ascribed to the pinning effects of nanoscale Y_2_O_3_ particles dispersed in the Cu matrix. The Cu-Y_2_O_3_ alloys with high strength and high thermal conductivity have potential applications in high thermal load components of fusion reactors.

## 1. Introduction

Copper alloys are the main candidate materials for the heat sink of the water-cooled target of a divertor in nuclear fusion reactors, due to its high thermal conductivity, strength and good radiation resistance [1,2,3,4,5]. Pure copper has a very high thermal conductivity but its strength is relatively low especially at high temperatures. Furthermore, the service life of pure copper is limited because of its high creep, swelling rate and irradiation hardening [6,7]. Therefore, it is necessary to develop copper alloys with both high strength and good thermal conductivity. 

CuCrZr alloys are the primary candidates for heat sink materials in the International Thermonuclear Experimental Reactor (ITER) due to its high thermal conductivity and high strength at medium and low temperature [8,9,10]. However, long-term thermal exposure of CuCrZr alloy to excessive temperature (>400 °C) leads to loss of strength due to over-ageing where the precipitates undergo Ostwald ripening losing their effective resistance as a barrier against dislocation glide [11]. Furthermore, neutron irradiation will cause degradation of thermal conductivity and mechanical properties of the alloy [12]. These drawbacks limit the operative temperature window of CuCrZr alloys. Developing a high-performance copper alloy to provide the best combination of excellent mechanical strength and good thermal conductivity at high temperatures is the main trend. Dispersion-strengthened copper alloys are an effective method to improve the high temperature strength of Cu-based materials. Highly stable ceramic particles (such as Al_2_O_3_ [11,13], TiC [14,15], NbC [16] and Y_2_O_3_ [17,18,19]) dispersed in the copper matrix can effectively hinder the motion of dislocations and migration of the grain boundary and extend the operating temperature range of copper alloys [20]. Among them, the Y_2_O_3_ dispersion-strengthened Cu alloys attract intense attention due to their excellent mechanical strength with higher thermal conductivity. The Cu-0.46wt.%Y_2_O_3_ alloy (Glidcop Al25) has been one of the Cu alloys considered for ITER [5,21]. 

Recently, Y_2_O_3_ dispersion-strengthened copper alloys have attracted increased attention. Compared with alumina, Y_2_O_3_ is relatively more suitable because of the low solubility of element Y in copper (less than 0.05 wt.% at 300 °C), which helps to maintain a high thermal conductivity [17]. In addition, the high thermodynamic stability of Y_2_O_3_ enhances the coarsening resistance at high temperatures [22]. Many efforts have been devoted to develop Y_2_O_3_ dispersion-strengthened copper alloys by Mechanical Alloying (MA) methods [6,7,17,18,23,24,25,26]. For example, G. Carro et al. [6] investigated the microstructural and mechanical properties of Cu-0.8wt.%Y produced by a powder metallurgy route and subsequent consolidation by hot isostatic pressing. The resultant equiaxed grain size distribution ranged from 0.5 μm to 50 μm, which greatly weakens the effect of fine grain strengthening. S.M.S. Aghamiri et al. successfully prepared a Cu-0.42wt.%Y_2_O_3_ alloy with comparable strength to the Glidcop-Al25 alloy by adding stearic acid as a process control agent and using MA and spark plasma sintering (SPS) [23]. Bing MA et al. explored the influence of Cu-Y compound content on the microstructure of Cu-Y_2_O_3_ alloys synthesized by MA and HIP process [26]. However, Y_2_O_3_ particles are prone to aggregation and growing up in the MA synthesized Cu-Y_2_O_3_ alloys, and impurities are easily introduced in the MA process. As the mechanical properties of dispersion-strengthened alloys are closely linked to the size and distribution of dispersoids, the segregation and large size of Y_2_O_3_ in copper alloys would inevitably reduce the mechanical properties. 

In this work, a citric acid sol-gel method [27] involving molecular level mixing was adopted to synthesize nanoscale Y_2_O_3_ particles homogeneously dispersed copper powders, and ultrafine-grained Cu-1wt.%Y_2_O_3_ alloy with high strength, high thermal conductivity and excellent thermal stability was obtained through spark plasma sintering (SPS) from sol-gel synthesized powders. The influence of Y_2_O_3_ nanoparticles on the mechanical properties, microstructure and thermal conductivity of the alloys was investigated and the corresponding mechanisms were discussed. 

## 2. Materials and Methods

Cu-1wt.%Y_2_O_3_ alloy powders were synthesized using a citric acid sol-gel method. Raw materials for the synthesis of Cu-1wt.%Y_2_O_3_ powders are listed in Table 1. The specific experimental process is as follows: C_6_H_8_O_7_·H_2_O was firstly dissolved in deionized water to create an acidic environment, then Cu(NO_3_)_2_·3H_2_O and YN_3_O_9_·6H_2_O were added into the solution. Citric acid is employed as chelating agent, which can provide the mixing of cations at the molecular level in the sol-gel process. The solution was heated at 80 °C with continuous stirring. An appropriate amount of polyethylene glycol (PEG, molecular weight 20,000) was added as surfactant after stirring at 80 °C for 2 h. The temperature was maintained at 80 °C with continuous stirring to remove excess water until a gel formed. The resulting gel was dried at 140 °C for 12 h and then was calcined at 550 °C for 6 h in air to remove the organic compounds, leading to a black mixture of CuO and Y_2_O_3_ powders. The calcined oxide powders were further ground to crush large particles, and subsequently, the oxides mixture was reduced in flowing hydrogen at 400 °C for 1 h, resulting in Cu-1wt.%Y_2_O_3_ powders. 

The sol-gel synthesized Cu-1wt.%Y_2_O_3_ powders were consolidated at 900 °C for 5 min under a pressure of 50 MPa using an SPS method to obtain bulk samples and the specific process can be seen in the literature [28]. The size of the sintered samples was 30 mm in diameter and about 3 mm in thickness. The densities of the samples were determined by the Archimedes principle using distilled water as the immersion liquid. The theoretical density of Cu-1wt.%Y_2_O_3_ alloy is 8.85 g/cm^3^ according to the rule of mixtures and the relative density of the sintered samples is about 97.6%.

The samples for the tensile tests were machined to a gauge dimension of about 5 × 1.5 × 0.75 mm^3^ using an electrical discharge machine and slightly polished. The tensile tests were carried out at room temperature (RT) with an Instron 5967 testing machine at a constant speed of 0.3 mm/min (corresponding to a nominal strain rate of 1 × 10^−3^ s^−1^) in air.

The thermal conductivity (γ) was calculated from the thermal diffusivity (α), density (ρ) and specific heat (C_p_) according to the relation: γ = αC_p_ρ. The α and C_p_ was determined using the laser flash diffusivity system (LFA457 Micro flash, NETZSCH). The sample was cut into cylinders with a diameter of about 12.6 mm and a thickness of about 2.5 mm and polished smooth with sandpaper. The thermal conductivity was measured at the Engineering Materials Science Experiment Center of University of Science and Technology of China (USTC, Hefei, China).

The sol-gel synthesized oxides precursor and reduced Cu-1wt.%Y_2_O_3_ powders were characterized by X-ray diffraction (XRD, X’Pert) with Cu Kα radiation. The morphology of the powders and tensile fracture of bulk samples was characterized by a field emission scanning electron microscope (FE-SEM, SU8020). Electron backscatter diffraction pattern (EBSD) mappings were collected using a Zeiss SIGMA field emission scanning electron microscope (SEM) equipped with a CRYSTAL detector (Oxford Instruments, Oxfordshire, UK) and the acceleration voltage was 13 kV. A misorientation angle of θ > 10° was used to distinguish the grain boundaries. The EBSD images were slightly filtered through the software (HKL Tango) to eliminate the influences of second phase particles on Cu grain observations. Microstructure characterization was carried out by a transmission electron microscopy (TEM, Tecnai G2 F20) at 200 kV to determine the size and distribution of Y_2_O_3_ particles in Cu-1wt.%Y_2_O_3_ alloys. Energy-dispersive X-ray spectroscopy (EDS, INCA) installed in the TEM was used for elemental analysis. The TEM samples were prepared by cutting flaked samples with diameters greater than 3 mm from the SPS sintered sample. First, the flakes were ground to about 50 μm in thickness. Subsequently, a Gatan 659 disk punch was used to cut a 3-mm-diameter disk from the thin wafer and then fixed on a molybdenum ring with an outer diameter of 3 mm. Then the specimen was transferred to Gatan 623 and further thinned to about 30 μm. Finally, the precision ion milling system (Gatan model 691) was used to polish the disk specimens at 4.3 keV to obtain electron transparency in the middle part. Note that the final angle and voltage of ion thinning for the TEM samples preparation in this study were 3° and 3.2 keV, respectively. 

## 3. Results and Discussion

### 3.1. Characteristics of Powders

**Figure 1:**
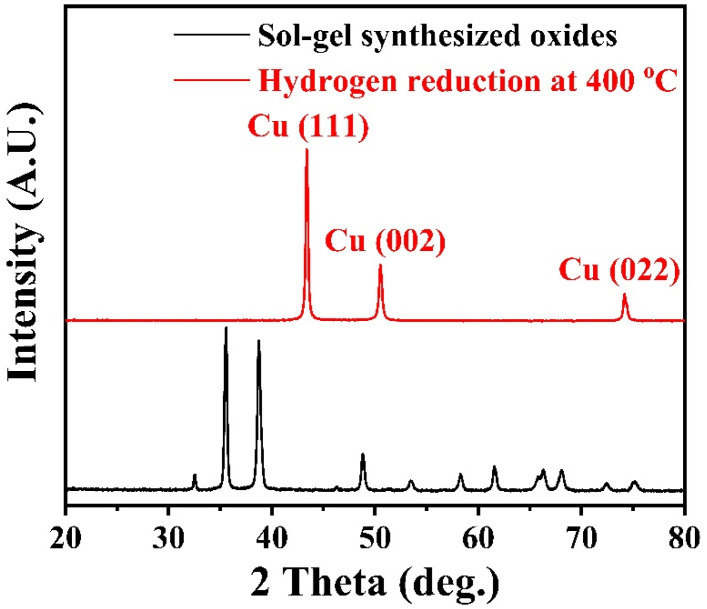
XRD patterns of sol-gel synthesized oxides and the powders hydrogen-reduction at 400 °C for 1 h.

**Figure 2:**
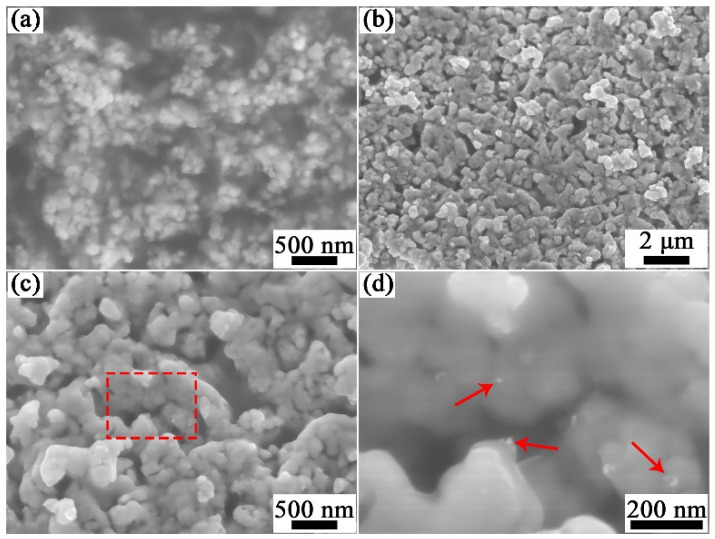
SEM images of (**a**) sol-gel synthesized precursor powders, (**b**,**c**) hydrogen reduced Cu-1wt.%Y_2_O_3_ powders at 400 °C for 1 h, and (**d**) magnification of the region marked with red dotted line in (**c**).

Figure 1 shows the XRD patterns of the sol-gel synthesized precursors before and after hydrogen reduction. The powder composition before hydrogen reduction is mainly composed of CuO and Y_2_O_3_ through software (Highscore plus 3.0) analysis. The XRD diffraction peaks at 35.55°, 38.67° and 48.84° correspond to the (11-1), (111) and (20-2) plane of CuO (PDF: 98-004-3179). After hydrogen reduction at 400 °C for 1 h, the XRD peaks at 43.33°, 50.46° and 74.15° corresponding to the (111), (002) and (022) plane of Cu (PDF: 98-062-7113) indicates that the CuO powders were completely reduced. The XRD diffraction peaks at 20.49°, 29.14° and 33.77° correspond to the (112), (222) and (004) plane of Y_2_O_3_ (PDF: 98-001-6394). However, no diffraction peak of yttrium oxide was found in XRD. This may be due to the low content and poor crystallinity of yttrium oxide, which will be further analyzed later in the powder TEM. Figure 2a shows the SEM images of the sol-gel synthesized precursor powders, consisting of CuO and Y_2_O_3_. The particle size distribution of the precursor powders is 20~200 nm with an average particle size of about 80 nm. After hydrogen reduction at 400 °C for 1 h, Cu particles grow up and sintering necks are formed between some Cu particles, as shown in Figure 2b,c. Figure 2d shows the high-magnification image of the rectangular region marked with a red dotted line in Figure 2c. Some nanoscale particles were evenly distributed on the surface and inside of the copper particles and the uniform distribution of these nanoparticles in copper powders is the prerequisite for obtaining high performance Cu-Y_2_O_3_ alloys.

**Figure 3:**
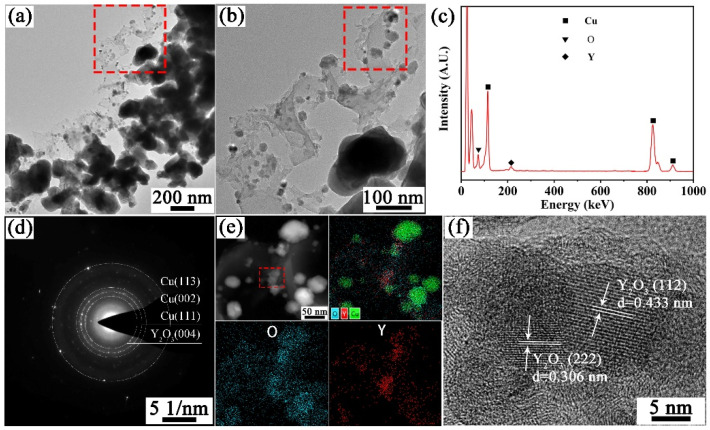
TEM images of hydrogen-reduced Cu-1wt.%Y_2_O_3_ powders at 400 °C for 1h, (**a**,**b**) TEM bright field images, (**c**) EDS results of the reduced powders, (**d**) SAED patterns of (**a**), (**e**) element mapping of the rectangular area in (**b**), and (**f**) HRTEM of Y_2_O_3_ particles indicated by the rectangle in (**e**).

Figure 3a,b shows the TEM images of the powders reduced at 400 °C for 1 h. Most of the particles are spherical, and some sintering necks were formed between the particles. Figure 3c shows the EDS results of Figure 3a, and the results show that there are three elements Cu, O and Y in the powders. Selected area electron diffraction (SAED) of Figure 3a is shown in Figure 3d; the result shows that the powders are composed of Cu and Y_2_O_3_. Elemental mapping analysis was performed on the rectangular region in Figure 3b to determine the composition of these particles, as shown in Figure 3e. The results show that most of these particles are Cu, while the distribution of Y and O elements and SAED results further confirm the existence of Y_2_O_3_. Figure 3f shows the high resolution TEM (HRTEM) image of the particles indicated by the rectangle in Figure 3e. The measured interplanar spacings of the two particles are 0.306 nm and 0.433 nm, respectively, corresponding to the (222) and (112) crystal planes of Y_2_O_3_ (PDF: 98-001-6394). 

### 3.2. Microstructure Characterization

**Figure 4:**
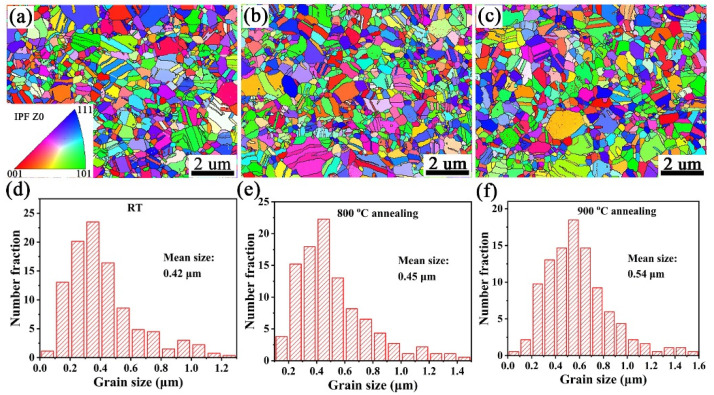
EBSD images of (**a**) SPSed Cu-1wt.%Y_2_O_3_ sample and the ones annealed at (**b**) 800 °C and (**c**) 900 °C, and (**d**–**f**) is the corresponding particle size distribution of (**a**–**c**).

The mechanical properties of dispersion-strengthened materials is closely linked to the grain structure and the size and distribution of the strengthening phases. Figure 4a,d shows the inverse pole figure (IPF) maps of as-sintered Cu-1wt.%Y_2_O_3_ alloy and the corresponding grain size distribution. The majority of Cu grains in the as-sintered Cu-1wt.%Y_2_O_3_ alloys are in the submicron range with an average grain size of 0.42 μm. Compared with the Cu-5vol%Y_2_O_3_ alloy (average grain size 1.15 μm) prepared by mechanical alloying [24], the grain size of the Cu-1wt.%Y_2_O_3_ alloy is obviously refined. After annealing at 800 °C, the grain growth in the Cu-1wt.%Y_2_O_3_ alloy is not obvious with an average grain size of 0.45 μm, as shown in Figure 4b,e. As the annealing temperature increases to 900 °C, the average grain size of the Cu-1wt.%Y_2_O_3_ alloy increases to 0.54 μm, and the annealed sample consist of two different types of grains: larger recrystallized grains and smaller submicron grains, as shown in Figure 4c,f. This phenomenon is attributed to the recrystallization of copper during high-temperature annealing. The recrystallized grains nucleate from the grain boundary and then many small submicron grains grow into recrystallized grains. Meanwhile, the Y_2_O_3_ particles distributed in the Cu matrix can effectively inhibit the growth of the grain and many grains are still smaller than 0.5 μm in size even after 900 °C annealing. Overall, the recrystallization temperature of Cu-1wt.%Y_2_O_3_ alloy samples is significantly higher than that of the Cu samples, and Y_2_O_3_ particles plays a significant role in the improvement of grain refinement and thermal stability. 

**Figure 5:**
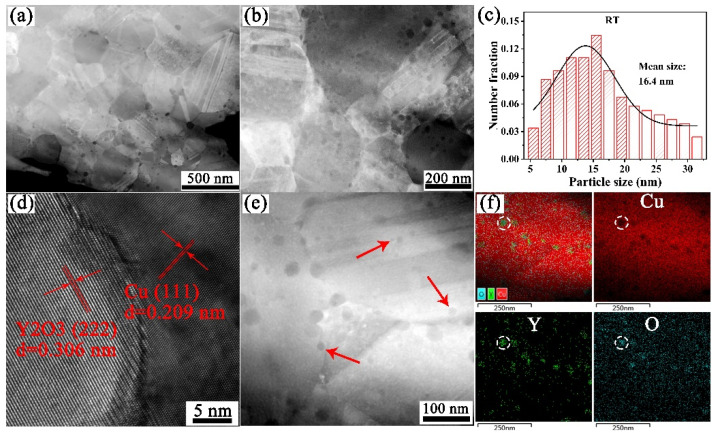
DF-STEM images (**a**,**b**), Y_2_O_3_ particles size distribution (**c**), HRTEM images of Cu and Y_2_O_3_ phase interface (**d**), and elemental distribution mapping (**e**,**f**) of Cu-1wt.%Y_2_O_3_ alloy.

In addition to the effect of grain size, the size and distribution of Y_2_O_3_ particles in the Cu-1wt.%Y_2_O_3_ alloy play an important role in improving the strength of the material. Figure 5 shows the dark field TEM (DF-TEM) images, particles size distribution of Y_2_O_3_ and elemental distribution mapping of the Cu-1wt.%Y_2_O_3_ alloy. It can be seen from the DF-TEM image in Figure 5a that there are many twins, most of which have grain sizes below 500 nm, and some larger Y_2_O_3_ particles are distributed at the grain boundaries. The specific distribution and size of Y_2_O_3_ particles on Cu are shown in Figure 5b,c. Nanoscale Y_2_O_3_ particles with an average size of about 16.4 nm are uniformly distributed in the interior and grain boundary of copper grains. Many Y_2_O_3_ particles with a particle size smaller than 10 nm are evenly distributed in the grains. Dispersing nanosized particles in the grain interior is an effective method to improve the strength and ductility, because the intragranular particles can generate, pin down and thus accumulate dislocations within the grains [29]. Figure 5d shows the high resolution transmission electron microscope (HRTEM) image of the interface between Y_2_O_3_ particles and the copper matrix. The measured interplanar distance of the Y_2_O_3_ particle and Cu matrix is 0.306 nm and 0.209 nm, respectively, corresponding to the values of the Y_2_O_3_ (222) and Cu (111) planes from the Powder Diffraction File (PDF) card of 98-001-6394 and 98-006-4699. In addition, the local area of particle distribution is analyzed through elemental distribution mapping, as shown in Figure 5e,f. Through the elemental distribution mapping of Cu, Y and O and analysis results of HR-TEM, it can be determined that the particles uniformly distributed in Cu matrix are Y_2_O_3_ particles. In fact, the uniform distribution and small size are attributed to the Cu-1wt.%Y_2_O_3_ alloy powders prepared using the citric acid sol-gel method and the short sintering time by SPS, which effectively reduces the number and size of Y_2_O_3_ particles at the grain boundaries. These Y_2_O_3_ nanoparticles with a high melting point and high hardness greatly improve the strength and thermal stability of the alloy. 

**Figure 6:**
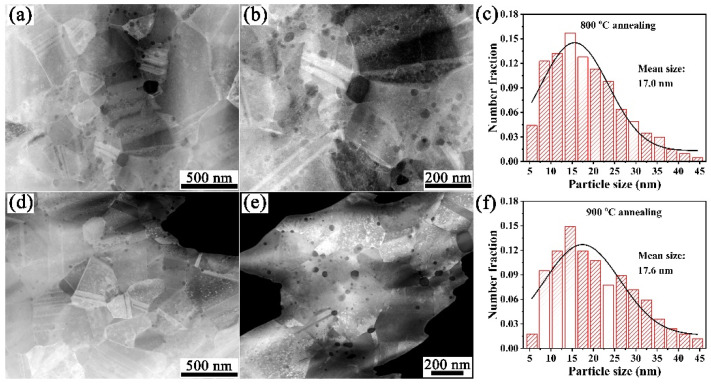
HAADF-STEM images and Y_2_O_3_ particles size distribution after annealing at different temperatures: (**a**–**c**) 800 °C annealing; (**d**–**f**) 900 °C annealing.

Figure 6 shows HAADF-STEM images and Y_2_O_3_ particles size distribution of the Cu-1wt.%Y_2_O_3_ samples annealed at 800 °C and 900 °C. After annealing at 800 °C, the average particle size of Y_2_O_3_ is 17.0 nm and the size and distribution of Y_2_O_3_ particles do not change significantly. After annealing at 900 °C, the grain size increased slightly to 17.6 nm. This phenomenon is consistent with the change of mechanical properties, which further explains the high thermal stability of the copper alloy.

### 3.3. Mechanical Properties

**Figure 7:**
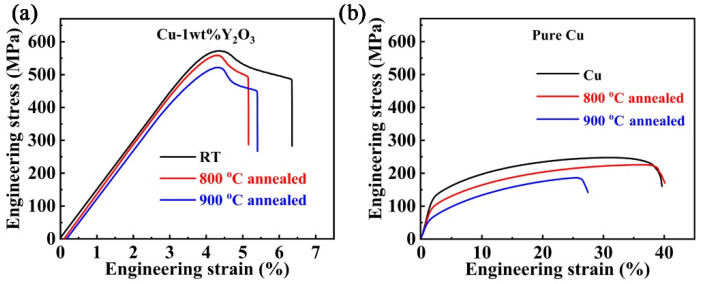
Tensile engineering stress-strain curves of (**a**) Cu-1wt.%Y_2_O_3_ and (**b**) pure Cu before and after annealing at different temperatures.

Figure 7 shows the engineering stress-strain curves of the Cu-1wt.%Y_2_O_3_ alloy and pure Cu annealed at 800 °C and 900 °C. The ultimate tensile strength (UTS) and total elongation (TE) of the alloy at RT are 572 MPa and 6.4%, respectively (Figure 7a). The strength is significantly improved compared with the pure Cu sample prepared by the same process (Figure 7b). In addition, compared with the Y_2_O_3_ dispersion strengthened copper alloy prepared by MA, the strength of the alloy has been greatly increased. For example, Zhou et al. reported Cu-5vol%Y_2_O_3_ alloys prepared by mechanical alloying, high temperature heat treatment and powder compact extrusion, with UTS and TE of 389 MPa and 8.9%, respectively [24]. The UTS of the present Cu-1wt.%Y_2_O_3_ alloys is similar to that of the Cu-60wt.%W composite prepared using the melt-infiltration method [30]. Compared with precipitation-strengthened CuCrZr alloy and dispersion-strengthened CuAl-25 alloy, the strength of the Cu-1wt.%Y_2_O_3_ alloy is obviously improved, while the ductility is decreased [31]. The low ductility of the Cu-1wt.%Y_2_O_3_ alloy may arise from the relatively high porosity (2.4%) and the particles distributed at the grain boundaries. The pores and intergranular Y_2_O_3_ particles would act as stress concentrators and cause localized plastic deformation [30]. After annealing at 800 °C, the UTS of Cu-1wt.%Y_2_O_3_ alloys had no obvious change with a high value of 563MPa and the TE was 5.2%. After annealing at 900 °C, the UTS decreased to 532 MPa, which may be caused by partial recrystallization at this temperature. In a word, Cu-1wt.%Y_2_O_3_ alloys prepared using sol-gel method have a high strength and good thermal stability. 

**Figure 8:**
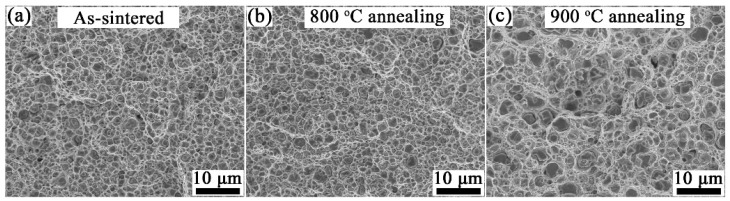
Fracture morphologies of (**a**) as-sintered Cu-1wt.%Y_2_O_3_ alloy and the samples annealed at (**b**) 800 °C and (**c**) 900 °C.

Figure 8 shows the tensile fracture morphologies of the as-sintered Cu-1wt.%Y_2_O_3_ and the ones annealed at 800 °C and 900 °C. Many tiny dimples can be seen in the fracture morphology of the SPSed Cu-1wt.%Y_2_O_3_ alloy, indicating ductile fracture. The size and distribution of dimples in the sample annealed at 800 °C is similar to that of the SPSed one (Figure 8b). For the sample annealed at 900 °C (Figure 8c), the dimples appear to be larger and deeper, which may be caused by the recrystallization of the alloy.

### 3.4. Thermal Conductivity

**Figure 9:**
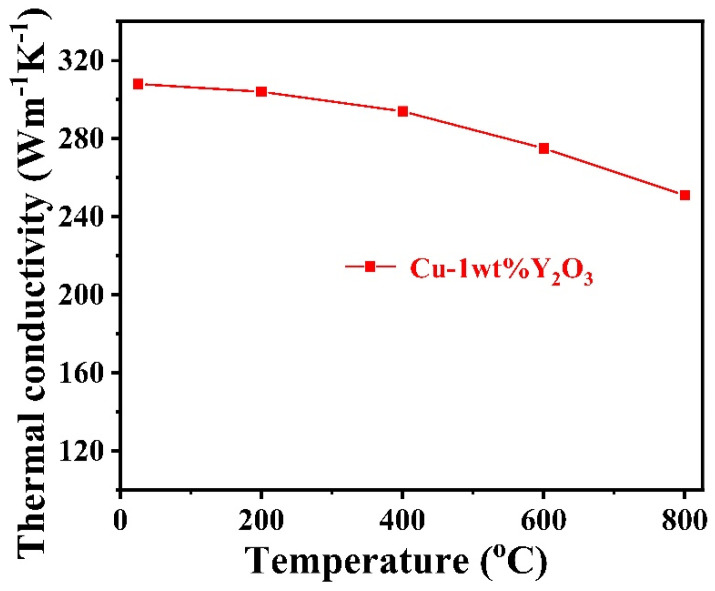
Thermal conductivity of Cu-1wt.%Y_2_O_3_ alloy at different temperatures.

The thermal conductivity of copper alloys is one of the most important parameters that determines whether they can be used as heat sink material for future fusion reactors. However, there are few mentions in the literature about the thermal conductivity of Y_2_O_3_ dispersion strengthened copper alloy. Figure 9 shows the thermal conductivity of the Cu-1wt.%Y_2_O_3_ alloy was measured at different temperatures from RT to 800 °C. The thermal conductivity of the Cu-1wt.%Y_2_O_3_ alloy at room temperature is 308 Wm^−1^K^−1^, which is lower than that of pure copper. The decrease in thermal conductivity after Y_2_O_3_ addition can be ascribed to the relatively lower thermal conductivity of Y_2_O_3_, and more defects such as the grain boundaries and Cu/Y_2_O_3_ phase interfaces than in pure Cu. These defects would scatter electrons and therefore reduced the thermal conductivity [28]. The thermal conductivity of the alloy decreases with the increasing temperature. Even so, the thermal conductivity at 800 °C of the Cu-1wt.%Y_2_O_3_ alloy is above 250 Wm^−1^K^−1^, which is still superior to the value of the W-Cu composite [32].

## 4. Conclusions

Nanostructured Cu-1wt.%Y_2_O_3_ alloy with high strength, high thermal conductivity and good thermal stability was fabricated by sol-gel synthesis and the SPS method. The sol-gel process involving molecular mixing can be used to prepare nanoscale Y_2_O_3_ homogenously dispersed copper powders. The average particle size of Y_2_O_3_ in Cu-1wt.%Y_2_O_3_ alloy is only 16.4 nm, which can effectively refine grains and improve the strength and thermal stability of the material by pinning dislocations and grain boundaries. The average grain size of a Cu-1wt.%Y_2_O_3_ alloy is about 0.42 μm. The tensile strength and thermal conductivity of the nanostructured Cu-1wt.%Y_2_O_3_ is 572 MPa and 308 Wm^−1^K^−1^, respectively. The strength and microstructure of the alloy do not change significantly after annealing at 800 °C, indicating excellent thermal stability. This work provides a promising method for fabricating high performance nanoparticle dispersion-strengthened Cu alloys.

## Figures and Tables

**Table 1:** Raw materials for the synthesis of Cu-1wt.%Y_2_O_3_ powders.

Raw Materials	Molecular Formula	Purity
Citric acid monohydrate	C_6_H_8_O_7_·H_2_O	≥99.5%
Copper nitrate trihydrate	Cu(NO_3_)_2_·3H_2_O	≥99%
Yttrium nitrate hexahydrate	YN_3_O_9_·6H_2_O	≥99.99%
Polyethylene glycol	HO(CH_2_CH_2_O)_n_H	-

## Data Availability

The raw/processed data of this work are available from the corresponding author upon reasonable request.
